# Targeting of Formyl Peptide Receptor 2 for *in vivo* imaging of acute vascular inflammation

**DOI:** 10.7150/thno.44226

**Published:** 2020-05-17

**Authors:** Tamara Boltersdorf, Junaid Ansari, Elena Y. Senchenkova, Jieny Groeper, Denise Pajonczyk, Shantel A. Vital, Gaganpreet Kaur, J. Steve Alexander, Thomas Vogl, Ursula Rescher, Nicholas J. Long, Felicity N. E. Gavins

**Affiliations:** 1 Department of Chemistry, Imperial College London, Molecular Sciences Research Hub, White City, London, W12 0BZ, UK.; 2 Department of Neurology, Louisiana State University Health Sciences Center-Shreveport, Shreveport, LA, 71130, USA.; 3 Department of Molecular & Cellular Physiology, Louisiana State University Health Sciences Center-Shreveport, Shreveport, LA, 71130, USA.; 4 Institute of Medical Biochemistry, Centre for Molecular Biology of Inflammation, University of Muenster, D-48149 Muenster, Germany.; 5 Institute for Immunology, University of Muenster, D-48149 Muenster, Germany.; 6 Department of Life Sciences, Brunel University London, Uxbridge, Middlesex, UB8 3PH, UK.

**Keywords:** Inflammation, neutrophils, formyl peptide receptors, small-molecule imaging probes, intravital microscopy

## Abstract

Inflammatory conditions are associated with a variety of diseases and can significantly contribute to their pathophysiology. Neutrophils are recognised as key players in driving vascular inflammation and promoting inflammation resolution. As a result, neutrophils, and specifically their surface formyl peptide receptors (FPRs), are attractive targets for non-invasive visualization of inflammatory disease states and studying mechanistic details of the process.

**Methods**: A small-molecule Formyl Peptide Receptor 2 (FPR2/ALX)-targeted compound was combined with two rhodamine-derived fluorescent tags to form firstly, a targeted probe (Rho-pip-C1) and secondly a targeted, pH-responsive probe (Rho-NH-C1) for *in vivo* applications. We tested internalization, toxicity and functional interactions with neutrophils *in vitro* for both compounds, as well as the fluorescence switching response of Rho-NH-C1 to neutrophil activation. Finally, *in vivo* imaging (fluorescent intravital microscopy [IVM]) and therapeutic efficacy studies were performed in an inflammatory mouse model.

**Results**: *In vitro* studies showed that the compounds bound to human neutrophils via FPR2/ALX without causing internalization at relevant concentrations. Additionally, the compounds did not cause toxicity or affect neutrophil functional responses (e.g. chemotaxis or transmigration). *In vivo* studies using IVM showed Rho-pip-C1 bound to activated neutrophils in a model of vascular inflammation. The pH-sensitive (“switchable”) version termed Rho-NH-C1 validated these findings, showing fluorescent activity only in inflammatory conditions.

**Conclusions**: These results indicate a viable design of fluorescent probes that have the ability to detect inflammatory events by targeting activated neutrophils.

## Introduction

Inflammation has been described as the “new cardiovascular risk factor” 1, playing a key role in the pathophysiology of a number of different diseases including myocardial infarction 2, stroke 3, sickle cell disease 4-6, rheumatoid arthritis 7 and cancer 8, 9. Neutrophils are the most abundant leukocytes in the blood and are the first responders in the host's defence against infection and/or tissue damage, acting as modulators of inflammation 10. In order to observe neutrophils, it is necessary to design effective, non-invasive imaging probes that can identify areas of inflammation and to provide mechanistic insights into pharmacotherapy for diseases.

Fluorescence microscopy is a well-established, pre-clinical tool which has high sensitivity and spatial resolution allowing biological systems and events, such as inflammation, to be investigated on a cellular and subcellular level 11-13. Although the design of targeted fluorescent probes that enable the visualization of cells (e.g. neutrophils) are widely reported, they often have a number of drawbacks including large size, poor photostability and, in some cases, restricted labelling sites, in particular when using peptide-based targeting groups 14, 15, all of which makes them unsuitable as diagnostic tools.

One particular target of current interest are the Formyl Peptide Receptors (FPRs). These seven-transmembrane G protein-coupled receptors (GPCR) are expressed on a variety of cells including neutrophils, with three receptors subtypes in humans: FPR1, FPR2/ALX (termed the lipoxin receptor and previously known as Formyl Peptide Receptor-Like 1 (FPRL1)) and FPR3 (previously termed FPRL2), nomenclature detailed in reference 16 and shown in Table S1). FPR2/ALX in particular has been described as a key receptor involved in inflammation resolution 17-19 and an absence of the receptor has been shown to contribute to progressive disease states 20. As a result, FPR2/ALX is an important target for biomedical research.

Methods to track neutrophils via the FPR family have previously focussed on preparing imaging agents for magnetic resonance imaging (MRI) 21, positron emission tomography (PET) 22, optical 23 and single-photon emission computed tomography (SPECT) 24, 25 from synthetic modifications of peptide-based targeting units that were derived from the FPR1 antagonist cinnamoyl-F-(D)L-F-(D)L-F (cFLFLF) scaffold 26, 27. These probes bind mainly via FPR1 and often suffer from poor target to background ratios as a result of the high hydrophobicity of cFLFLF, which necessitates incorporation of for instance, large, PEGylated chains. In addition, their preparation is expensive and peptide-based agents are thought to be less synthetically and metabolically robust than non-peptidic ligands 28. Similarly, the potent pan-FPR agonist fMLF (formyl-Met-Leu-Phe) has been modified by solid state peptide synthesis strategies to prepare probes for both optical and radio-imaging 29-31 and a probe based on a peptidic FPR2/ALX agonist, WKYMVm, has been used in microscopy 32. However, the risk with modifying potent receptor agonists to produce targeted imaging systems is unwanted receptor activation. To circumvent these issues, a non-peptidic, FPR2/ALX agonist 4-butoxy-N-[2-(4-methoxy-phenyl)-4-oxo-1,4-dihydro-2H-quinazolin-3-yl]-benzamide (Quin C1), that has been reported to exhibit concentration dependent properties 33, 34 was considered. The agonistic effect of Quin C1 on neutrophils is only observed at higher concentrations compared to previous peptide-based counterparts such as fMLF or CFLFLF^22^, ^35^, and synthetic modifications on the Quin C1 amine are possible, making it an ideal target for probe design.

Based on these findings, we have recently reported a combination of this small-molecule with metal complexes to provide long-lived luminescent signals for time-resolved detection in fixed cells and found that the compound was preferentially taken up in activated neutrophils 36. However, these metal-based compounds were not applicable for *in vivo* applications due to high concentrations required for visualization in a biological setting. In the current work, we have expanded these findings to develop, prepare and characterize a derivative of Quin C1 to form an FPR2/ALX-targeted imaging probe which can be used for tracking inflammation *in vivo*. For this purpose, a rhodamine B-derived fluorophore was chosen due to well-established compatibility with biological applications, high quantum yields, good stability and ease of synthetic modification 37. Additionally, Rhodamine B dyes emit in the low energy visible region and as a result, provide a convenient compromise for preclinical imaging between providing the level of tissue penetration (i.e. long enough wavelengths) required for *in vivo* studies while at the same time maintaining some resistance to decomposition and photobleaching compared to NIR dyes 38. Via synthetic modifications, we sought to prepare one motif which excludes the possibility of rhodamine spirocyclisation 37 (which results in formation of a non-emissive species) to act as a probe for neutrophils under inflammatory conditions and constructed a second, “switchable” (pH-sensitive) analogue, which remains non-fluorescent under normal physiological conditions but switches “on” when neutrophils have been stimulated. We herein demonstrate the utility of Rho-pip-C1 as a probe to visualize acute inflammation via neutrophil targeting along with establishing a pH-sensitive probe which specifically “switches on” under inflammatory conditions. Thus, our novel probes enable the visualization of inflammation for biomedical research and can be used as screening tools for drug discovery.

## Material and Methods

### Materials

All commercially available reagents were bought from Sigma Aldrich (St-Louis, MO, USA) or Fisher Scientific (San Diego, CA, USA) unless otherwise stated.

### Drugs and antibodies

Tumor necrosis factor-alpha (TNFα, 250 ng per 200 µL) 39 R&D systems (Minneapolis, MN, USA) and BMS-470539 (20 mg/kg IP, Tocris Bioscience, Bristol, UK; batch No: 1A/156791) 40 were used for *in vivo* studies. Vehicle, phosphate buffered saline (PBS, Life Technologies, Grand Island, NY, USA), Boc2 (10 µM) (N-*tert*-butoxycarbonyl-L-Phe-D-Leu-L-Phe-D-Leu-L-Phe, MP Biomedicals, Cambridge, UK) and WRW4 (10 µM) (Trp-Arg-Trp-Trp-Trp-Trp, EMD Biosciences Inc, San Diego, CA, USA) were used as FPR antagonists 41. Phorbol myristate acetate (PMA, 100 µM) was used to stimulate neutrophils 42. Leukotriene B_4_ (LTB_4_) (1 µM) was used as a chemoattractant for chemotaxis and transmigration assays.

### General synthetic procedures

Deuterated solvents were bought from Goss Scientific. ^1^H-NMR and ^13^C-NMR spectra were recorded on a Bruker AMX-400 spectrometer at room temperature unless stated otherwise and fully assigned where possible using 2D correlation spectroscopy. Coupling constants are quoted in Hz and multiplicities are abbreviated as: s = singlet, d = doublet, t = triplet, m = multiplet and br = broad. Electrospray Time-of- Flight and MALDI mass spectra were obtained using a Waters LCT Premier. Thin-layer chromatography was conducted using pre-coated Silica gel 60, F254 plates with a thickness of 0.2 nm. UV-vis absorption spectra were recorded on a Perkin Elmer 650 spectrometer and fluorescence spectra were obtained using a Varian Cary Eclipse spectrophotometer. For pH measurements a Jenway model 3510 pH/mV/temperature meter was used calibrated against pH 10.00, pH 7.00 and pH 4.00 buffers. Column chromatography was performed using silica gel and laboratory grade solvents, under mild pressure. Synthetic and spectral details are given in the supplementary information.

### Animals

Male C57BL/6 mice were purchased from Jackson Laboratory (Bar Harbor, ME) at 8-10 weeks of age (28-35 g). Mice were maintained on a 12 hours (h) light-dark cycle during which room temperature was maintained at 21-23 °C and had access to a standard chow pellet diet and tap water ad libitum. All animal experiments were approved by the Louisiana State University Health Sciences Center-Shreveport (LSUHSC-S) Institutional Animal Care and Use Committee (IACUC) and were in accordance with the guidelines of the American Physiological Society, or complied with ARRIVE (Animal Research: Reporting In Vivo Experiments) guidelines and followed the European Union Directive (2010/63/EU). All studies were performed blinded and randomized, with a key system to identify which animal/sample had undergone which treatment. Furthermore, compounds administered were made by laboratory personnel other than the one performing the experiment.

### Human samples

The study was approved by the institutional review board of the LSUHSC-S (STUDY00000261) and conducted in accordance with the Declaration of Helsinki. After signed consent was obtained, blood was taken from control volunteers (23 - 65 years old. Eleven males, four females). Volunteers that were pregnant were excluded from the study.

### Neutrophil isolation and treatment

Blood was collected from healthy volunteers after obtaining informed consent in 50 mL syringes that had already been filled with 5 mL acid citrate dextrose (ACD) solution. Blood was transferred evenly into two 50 mL tubes and spun at 1000 rpm for 20 minutes at room temperature (with brakes at 6:6 to avoid neutrophil activation). The plasma layer was removed and blood was separated with a double-density gradient using 6% dextran (incubate for 15- 20 minutes and collect leukocyte layer), followed by Histopaque 1077 43. After centrifugation at 1500 rpm for 30 minutes, neutrophils were collected as a pellet and contaminating red blood cells were removed by osmotic lysis in ice-cold water. PMN cells were washed in PBS and re-suspended in Dulbecco's modified Eagle's medium (DMEM) with 3% fetal calf serum (FCS. Biochrome, Cambridge, UK).

### *Ex vivo* generation of murine neutrophils from Hoxb8 conditionally immortalized neutrophil progenitors

The Hoxb8 neutrophil model was generated and used essentially as described 44. In brief, Hoxb8-dependent neutrophil progenitors were generated by transducing progenitor cells enriched from C57BL/6 bone marrow single cell suspensions with a murine retroviral expression vector for the transcription factor Hoxb8 fused to the human estrogen receptor (Hoxb8-ER). The resulting immortalized line was kept in Optimem Glutamax medium (Fisher Scientific) supplemented with 100 U/mL penicillin, and 0.1 mg/mL streptomycin, 30 µM beta-mercaptoethanol, 10% fetal bovine serum (FBS) (Biochrome, Cambridge, UK), 20 ng/mL recombinant SCF (Immunotools, Friesoythe, Germany), and 1 µM beta-estradiol. Neutrophil differentiation was induced by estrogen withdrawal for 4 days and was monitored by flow cytometry for complete downregulation of c-Kit and CD34, and upregulation of CD11b and Ly6G. Cells were cultured at 37°C in in a 5% CO_2_ atmosphere.

The human promyelocytic cell line HL-60 was cultivated in RPMI-VLE (Millipore, Burlington, MA, USA) supplemented with 1% L-glutamine, 1% non-essential amino acids, 100 U/mL penicillin, and 0.1 mg/mL streptomycin, and 10% FBS and was induced to differentiate toward neutrophil-like cells for seven days via addition of 1.25% DMSO (Applichem, Darmstadt, Germany). Cells were cultured at 37 °C in a 5% CO_2_ atmosphere. Expression of FPR1 and FPR2 upon differentiation was confirmed by qPCR using the Quantitect (Qiagen, Hilden, Germany). HeLa cells stably expressing N-terminally FLAG-tagged human FPR1 or FPR2/ALX were cultured in DMEM and supplemented with 10% FBS, 100 U/mL penicillin, and 0.1 mg/mL streptomycin. Cells were cultured at 37 °C in in a 5% CO_2_ atmosphere**.**

### Cytotoxicity analysis

Cells were treated with the indicated agonists for 15 minutes at 37 °C and were then washed and resuspended in Cell Wash (BD Bioscience) containing 50 nM of the cell-impermeant far-red emitting nucleic acid stain Helix NP NIR (Biolegend, San Diego, CA, USA) for detection of compromised cells. A Guava easyCyte™ System (Millipore, Burlington, MA, USA) was used to determine the percentage of Helix NP NIR-negative cells per 10,000 cells analysed. Triton X-100 (1%) was used as a positive control.

### Detection and quantification of reactive oxygen species (ROS) generation

ROS production was measured by flow cytometry using dihydrorhodamine 123 (DHR123). Cells were preincubated for 20 minutes at 37 °C. The indicated agonists and DHR (2 µM) were then added for 15 minutes. To stop the reaction, cells were placed on ice, washed, and resuspended in Cell Wash (BD Bioscience) containing 0.5% PFA and 50 nM of the cell-impermeant far-red emitting nucleic acid stain Helix NP NIR (Biolegend) to gate for live cells. 10,000 live cells per condition were analysed on a Guava easyCyte™ System (Millipore, Burlington, MA, USA) for median fluorescence intensity. Agonist-mediated ROS production was expressed as percentage of the signal detected in untreated cells. The FPR2/ALX specific agonist WKYMVm was used as a positive control.

### Chemotaxis assay

For the chemotaxis assay, neutrophils were diluted to a concentration of 4 × 10^6^ cells/mL in DMEM and 3% FCS and then treated with different concentrations (10^-6^ M to 10^-9^ M) of both the compounds for 10 minutes. The assay was performed using 3 μm pore size ChemoTx® System 96 well plates (Neuro Probe, Gaithersburg, MD USA) by adding 29 μL of 10^-6^ M LTB_4_ as a chemotactic stimulus or PBS as control to the bottom wells, and 25 μL of neutrophil-vehicle/compound suspension on top of the filter membrane. Plates were incubated for 3 hours at 37 °C with 5% CO_2_. After 3 hours the filter membrane was removed, and migrated cells were manually counted by taking 20 μL from the bottom wells and diluting in a 1:1 ratio with trypan blue. Results were expressed as an average of total number of cells in the bottom well from samples run in triplicate.

### Transmigration assay

For the transmigration assay, neutrophils were diluted to the concentration of 1 × 10^6^ cells/mL in DMEM with 3% FCS and then treated with different concentrations (10^-6^ M to 10^-9^ M) of the compounds for 10 minutes. Prior to this step, 24-well plate inserts were treated with fibronectin for 30 minutes. Next, 32,000 human umbilical vein endothelial cells (HUVECs) cells/200 μL were added and grown for 72 hours to form a confluent monolayer. When the monolayer had formed, HUVEC media was removed and 500 μL of treated neutrophil-vehicle/compound suspension was added onto the inserts. In a fresh 24-well plate 500 μL of PBS (control) or chemoattractant (10^-6^ M LTB_4_) were added and inserts were transferred to the plate. Plates were incubated for 3 hours at 37 °C with 5% CO_2_. After 3 hours the inserts were removed, and migrated cells were manually counted by taking 20 μL from the bottom wells and diluting in a 1:1 ratio with trypan blue. Results were expressed as total number of cells in the bottom well.

### Myeloperoxidase release assay (MPO)

For the MPO release assay, three solutions were prepared; solution 1 consisted of KH_2_PO_4_ (5.4 g, 0.040 mol)_,_ EDTA (1.875 g, 0.006 mol) and Triton-x-100 (5 ml) at pH 5.4. Solution 2 was made up of 3,3',5,5'- tetramethylbenzidine (TMB, 10 mM) prepared in acetone (2 ml) and solution 3 contained 30% H_2_O_2_ (25 µl) in H_2_O (10 ml). The final MPO assay solution was prepared by combining solutions 1, 2 and 3 in the ratio of 9:1:0.1 respectively. Neutrophils (1x 10^5^/well) were seeded in a 96 well plate and were treated with vehicle (PBS) or PMA for 3 hours at 37 °C, 5% CO_2_. In some cases, neutrophils were treated with different concentrations (10^-6^ M to 10^-9^ M) of the compounds for 10 minutes prior to PMA stimulation for 3 hours.

The neutrophil supernatant was collected from the 96 well plate (50 µl) and added to the well containing MPO solution (200 µl) (4:1 ratio), followed by incubation at room temperature until the color of the solution changed to blue. Addition of H_2_SO_4_ (25 µl, 1 M) terminated the reaction and the absorbance was read at 450 nm and presented as an average from samples run in triplicate.

### Immunofluorescence microscopy

Neutrophils (2 x 10^5^ cells per well in 200 μL) were seeded on poly-L-lysine coated coverslips (Discovery Labware, Bedford, MA, USA) in a 24 well plate and either stimulated with PMA (100 nM) for one hour or treated with vehicle (1x PBS) at 37°C, 5% CO_2_. Pharmacological blocking was carried out by waiting 30 minutes after seeding cells for equilibration before treating them with vehicle (saline), Boc2 (pan-FPR antagonist, 10 μM), or WRW4 (FPR2/ALX antagonist, 10 μM) at 37 °C, 5% CO_2_ for 15 minutes. The cells were then stained with Rho-pip-C1 or Rho-NH-C1 (1 μM) for another hour and fixed in 10% formalin. For cell identification, DNA was stained with the nuclear dye 4′,6-diamidino-2-phenylindole (DAPI; Molecular Probes, Eugene, OR, USA) in PBS for 10 minutes. After mounting (Fluoromont-G, Southern Biotech, Birmingham, Alabama, USA) the neutrophil-containing coverslips on glass slides, they were dried and firmly affixed using nail polish. The images were visualized using a Nikon Eclipse Ti inverted epifluorescence microscope (Minato-ku, Tokyo, Japan). In each experiment pictures were taken from three to four random fields with at least 30 cells per field of view 45. Relative fluorescence intensities were then quantified from every image within each donor sample by calculating the ratio between emission intensity in the in-built Texas red channel to assess cell-associated Rho-pip-C1 (λ_exc_ = 595 nm/ λ_em_ = 645 nm) and emission intensity in the in-built DAPI channel (λ_exc_ = 356 nm/ λ_em_ = 460 nm) was used as a read-out for cell numbers using software on NIS Elements Advanced Research (Nikon Instruments Inc, Melville, NY, USA). The data was averaged to give the final results.

### Internalization Assays

Flow cytometric analysis of receptor surface-associated immunofluorescence was used to determine agonist-induced internalization. Cells were left untreated or were incubated with Quin C1, or the derivatives Rho-pip-C1 and Rho-NH-C1 at the indicated concentrations for 15 minutes at 37 °C. W-peptide served as a positive control. Cells were incubated with 5 mM EDTA in PBS^-/-^ for 3 minutes at 37 °C and centrifuged at 200 x g for 5 minutes at 4 °C. To detect cell-surface accessible FLAG signal, cells were first incubated with ice-cold wash solution (PBS^-/-^, 5% BSA and 1 mM CaCl_2_) for 15 minutes on ice, followed by incubation for 45 minutes with 5 µg/mL DyLight488 (Thermo Fischer Scientific, San Diego, CA, USA)-conjugated anti-FLAG M1 antibody on ice. Cells were washed twice in ice-cold cell wash solution (BD Bioscience). Median fluorescence intensity of 10,000 cells per condition was measured using a Guava easyCyte™ System (Millipore, Burlington, MA, USA). Far-red emitting Helix NP NIR (Biolegend, San Diego, CA, USA) was used to exclude compromised cells. Dylight 488-conjugated mouse IgG2 was used as an isotype control. Internalization was measured via loss of cell surface signals in agonist-treated cells and was expressed as percentage of the signal detected in untreated samples. For each condition, at least six independent biological experiments were performed.

### Drug Treatment for Spinning Disc Intravital Microscopy imaging

Saline or recombinant mouse TNFα (250 ng in 200 µL saline) were injected via intrascrotal injection 2 hours before cremaster surgical procedure 39. A separate group of mice were treated with BMS-470539 (20 mg/kg) via intraperitoneal (i.p.) injection, 1 hour post TNFα injection 40. No mortality occurred.

### Spinning Disc Intravital Microscopy

Mice were anesthetized with ketamine hydrochloride (150 mg/kg body weight) i.p. and xylazine (7.5 mg/kg body weight i.p.) and the jugular vein was cannulated for drugs/antibody administration). Rho-pip-C1 (15 µg in 50 µL of saline) was injected for recirculation one hour before leukocyte (neutrophil) observation. The cremaster muscle was prepared for intravital microscopic observation as previously described 46. Body temperature was maintained at 35-37 **°**C. After a 20 minute stabilization period, the cremasteric microcirculation was visualized using a spinning disc intravital microscope (an Olympus BX51WI upright microscope with a 40XW (LUMPLFLN) (Olympus, Center Valley, PA, USA) objective equipped with a 3i LaserStack laser launch (3i, Denver, CO, USA), Yokogawa CSU-X1-A1N-E spinning disk confocal unit (Yokogawa Electric Corporation, Musashino, Tokyo, Japan) and electron multiplier CCD camera (C9100-13; Hamamatsu, Bridgewater, NJ, USA)). Alexa Fluor 488 conjugated anti-mouse Ly-6G (Gr-1) clone RB6-8C5 (Biolegend, San Diego, CA, USA) (2 µg/mouse) was used to identify neutrophils. 5-11 venular segments (diameter 20-50 µm) were selected for evaluation and recorded. The number of adherent (stationary for ≥ 2 seconds) and extravasated (the number that had extravasated up to 50 μm either side of the 100 μm vessel length) neutrophils per vessel segment were measured and averaged for each animal. Data was analysed offline using Slidebook software (3i, Denver, CO, USA).

### Statistical Analysis

All data was tested to follow a normal distribution using Kolmogorov-Smirnov test of normality with Dallal-Wilkinson-Lillie for corrected *p* value. Data that passed the normality assumption was analyzed using Student's *t*-test (two groups) or ANOVA with Bonferroni post-tests (more than two groups). Data that failed the normality assumption were analyzed using the non-parametric Mann-Whitney U test (two groups) or Friedman with Dunn's test (more than two groups). Analysis was performed using Graph Pad Prism 6.0 or 8.0 software (San Diego, USA). Data are shown as mean values ± standard error of the mean (SEM). Differences were considered statistically significant at a value of *p* < 0.05. The figures have been graphically presented on a range-specific axis. All data and statistical analysis comply with Theranostics guidelines 47.

## Results

### Preparation of a small-molecule FPR2/ALX fluorescence probe (Rho-pip-C1)

**Figure 1:**
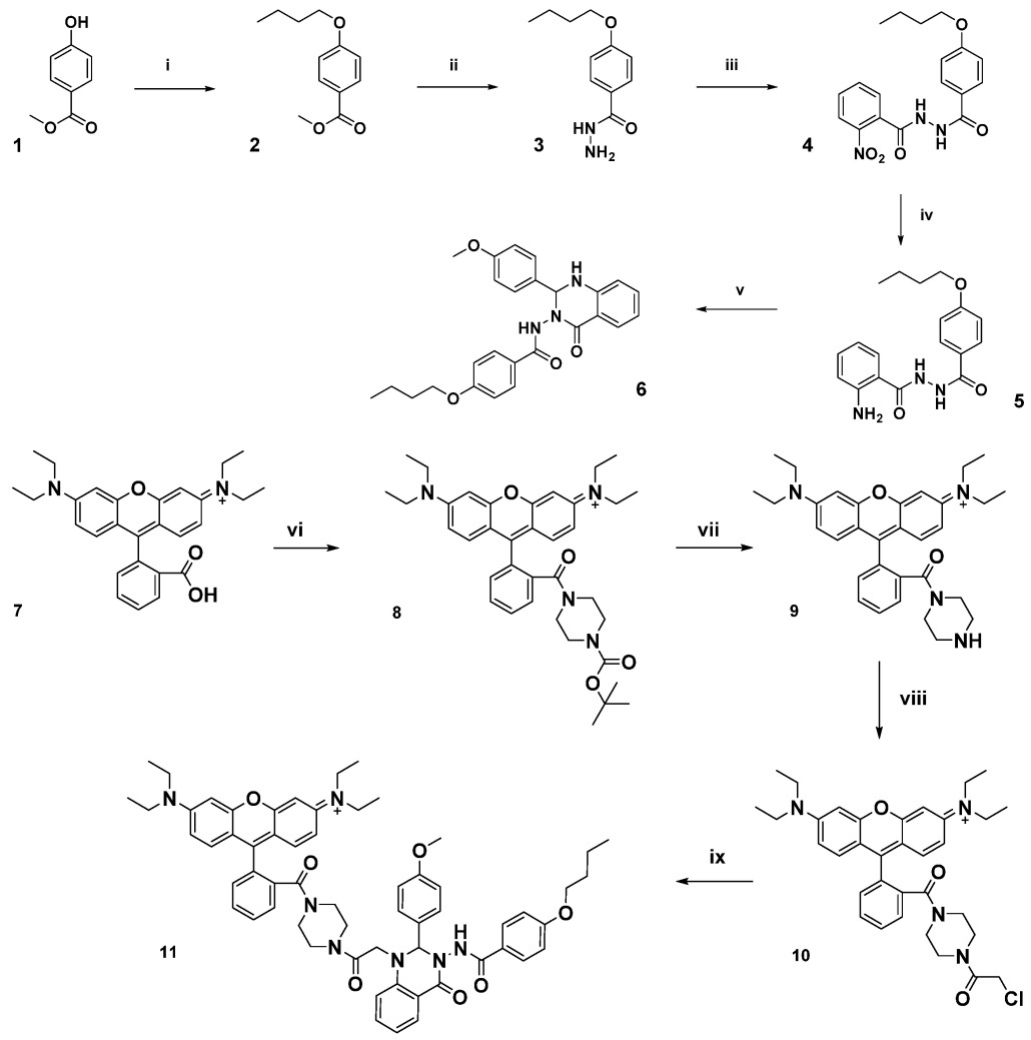
** Schematic of synthetic pathway for preparation of FPR2/ALX-targeted ligand (6) and the functionalized fluorescent probe (11, Rho-pip-C1).** Reagents: i) 1-bromobutane, potassium carbonate, methanol, ii) hydrazine monohydrate, ethanol, iii) 2-nitro-benzoyl chloride, potassium carbonate, dichloromethane, iv) zinc dust, acetic acid, dichloromethane, v) 4-methoxy benzaldehyde, citric acid, ethanol, vi) *tert*-butyl piperazine-1-carboxylate, triethylamine, N,N,N′,N′-tetramethyl-O-(1H-benzotriazol-1-yl)uronium hexafluorophosphate and dichloromethane, vii) trifluoroacetic acid, dichloromethane, viii) chloroacetyl chloride, triethylamine, dichloromethane ix) 6, N,N-diisopropylethylamine, acetonitrile.

The FPR2/ALX targeting unit Quin C1 **(6)** 33, identified from an FPR2/ALX binding compound library screening hit, was prepared from commercially available 4-hydroxymethyl benzoate as previously reported (Figure 1) and combined with a fluorescent dye 36. The dye of choice, a rhodamine B derivative, is known for a cyclization equilibrium which is characterized by a closed, nonfluorescent spirolactam or lactone form, which is almost colourless 37, 48. At low pH values, the carbonyl group becomes activated and a fluorescent, fully conjugated, ring-opened analogue is formed. This “switchable” property has been widely investigated in the sensing of heavy metal ions 49 and for preparation of pH-sensitive analytic probes 50.

Initially, to prepare a non-switchable, fluorescent Quin C1-rhodamine conjugate, 1,4-piperazine-BOC was appended to rhodamine B (**7**) in a peptide coupling reaction to form a tertiary amine (**8**) and thus lock the rhodamine spirocyclic unit into its ring-opened, fluorescent state (see Figure 1 and Supplemental Material for synthetic procedures, Figures S1-4 LC chromatograms). Subsequent deprotection of the *tert*-butyl group, followed by addition of chloroacetyl chloride resulted in acylation of the secondary amine (**10**). Finally, Quin C1 was appended on the aromatic amine functional site to yield **11** (termed Rho-pip-C1). As expected, photophysical properties were not found to be significantly pH-dependent and a strong, rhodamine-derived emission band at 582 nm was observed in methanol upon excitation at 350 nm, as well as overlapping, broad maxima at 403 nm, 428 nm and 453 nm (Figure S5).

### Rho-pip-C1 does not cause cytotoxicity

Initially we examined the cytotoxicity of Quin C1, Rho-pip-C1 and Rho-NH-C1 in both human neutrophilic-differentiated HL60 cells and murine neutrophils produced from Hoxb8-dependent neutrophil progenitors. As shown in Figure S6 no cytotoxicity was observed for Quin C1, Rho-NH-C1 or Rho-pip-C1 at the concentrations tested (10^-6^ M - 10^-9^ M), whereas the majority of the cells were compromised after 15 minutes exposure to Triton X-100, included as a positive control.

### Rho-pip-C1 does not induce internalization of FPR2/ALX

**Figure 2:**
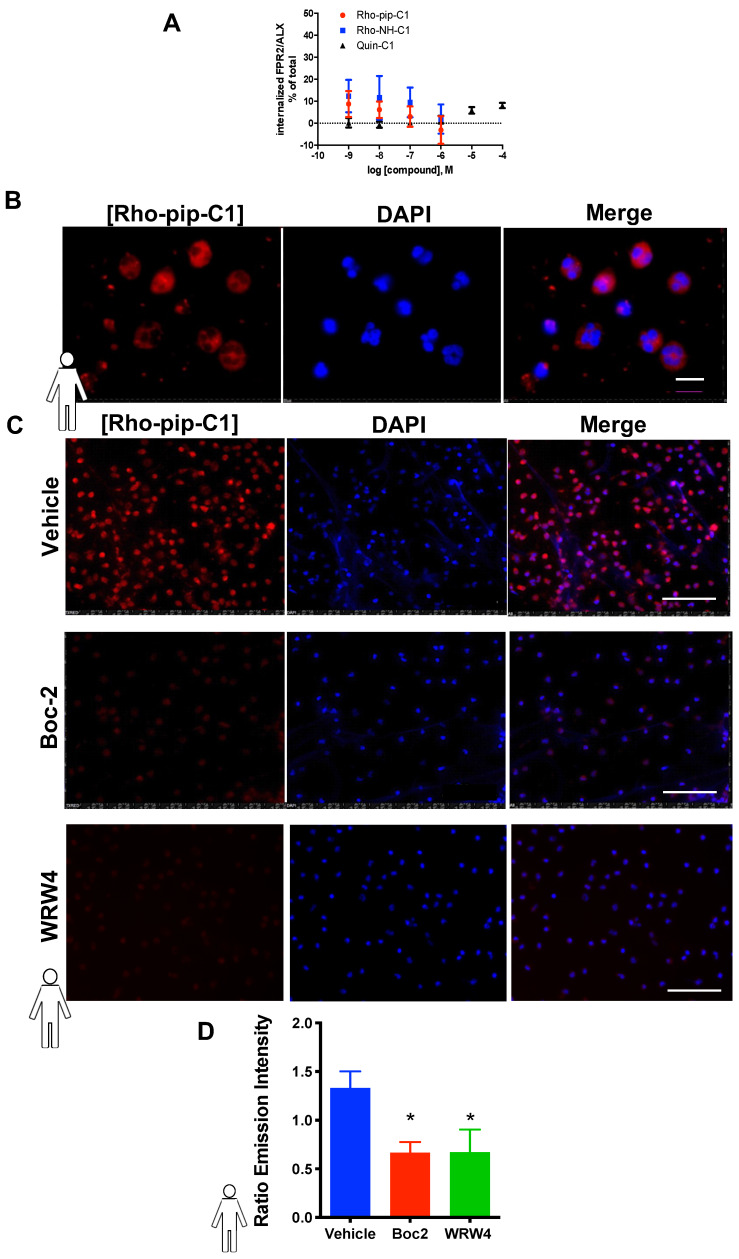
** Rho-pip-C1 binds to FPR2/ALX, without causing receptor internalization.** (A) Flow-cytometric analysis of FPR2 internalization. HeLa cells stably expressing N-terminally FLAG-tagged human FPR2/ALX were used to determine whether Rho-pip-C1 and Rho-NH-C1 induce FPR2/ALX internalization at a concentration range of 10^-6^ to 10^-9^M. Results are expressed as a loss of FPR2/ALX upon agonist treatment relative to untreated cells. The lead compound Quin C1 was included for comparison. (B) Representative magnified images (100X) of fixed human neutrophils treated with Rho-pip-C1 and the nuclear stain DAPI (scale bar, 10 μM). (C) Representative immunofluorescence images from n = 5 independent donors in cells that had been treated with vehicle (PBS), the pan-FPR antagonist Boc2 (10 µM), or the FPR2 specific antagonist WRW4 prior to incubation with Rho-pip-C1 (scale bar, 100 μM). (D) Quantification of emission intensity ratios for vehicle (PBS), Boc2 (10 µM) and the FPR2/ALX specific antagonist WRW4 (10 µM) (n = 5 independent donors in each group). All imaging analysis was done in a double-blinded fashion. Statistical significance was determined using one-way ANOVA with Bonferroni post-hoc test and is presented as *p < 0.05 vs. PBS control.

It is known that Quin C1 functions as a *selective* agonist for the FPR2/ALX^34^. G-protein coupled receptors (such as FPR2/ALX) rapidly internalize upon ligand binding, which is not advantageous in a tracer. However, Quin C1 elicits FPR2/ALX internalization only at concentrations above 1 µM 33. To assess whether Rho-pip-C1 had any effect on receptor internalization, we examined whether the compound was able to stimulate FPR2/ALX internalization using HeLa cells stably expressing N-terminally FLAG-tagged human FPR2/ALX 51. Figure 2A shows that no significant changes in receptor internalization were observed over the chosen concentration range (10^-6^ to 10^-9^ M) between Quin C1 and Rho-pip-C1, supporting the concept that Rho-pip-C1 is a suitable imaging probe as ideal probes should not elicit internalization.

### Rho-pip-C1 labels neutrophils via binding to FPR2/ALX

To assess whether the Quin C1-derived probe Rho-pip-C1 could be used as a neutrophil label, neutrophils were isolated and incubated with Rho-pip-C1. As shown in Figure 2B and 2C, neutrophils incubated with Rho-pip-C1 displayed bright fluorescence labelling. These results were confirmed in a quantitative manner by measuring fluorescence emission intensities of cell-associated Rho-pip-C1 (Figure 2C). To verify whether Rho-pip-C1 was producing its fluorescence signal via the FPR family, neutrophils were treated with the pan-FPR antagonist Boc2 prior to Rho-pip-C1. Figure 2D shows that blocking of the FPRs with the pan antagonist Boc2 induced a significant decrease (*p* < 0.05) in the neutrophil-associated signal intensity. To further confirm whether the observed effects were specifically due to the actions of Rho-pip-C1 binding to FPR2/ALX, we repeated the experiments in the presence of the FPR2/ALX specific antagonist WRW4. Selective antagonism of neutrophil FPR2/ALX reduced the neutrophil Rho-pip-C1 signal intensity, confirming that Rho-pip-C1 binds to FPR2/ALX, similar to Quin C1 (a known FPR2/ALX agonist).

### Rho-pip-C1 does not alter the ability of neutrophils to perform their normal physiological functions

**Figure 3:**
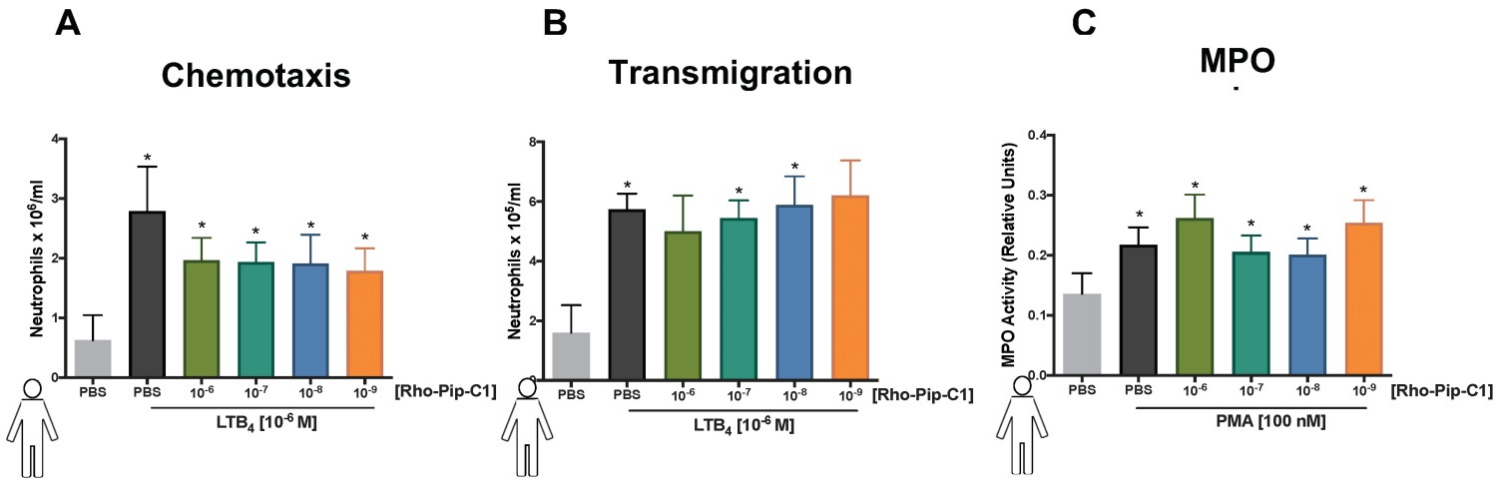
** Rho-Pip-C 1 does not elicit changes in neutrophil function.** (A) Neutrophils isolated from peripheral blood of healthy volunteers were resuspended in DMEM with 3% FBS and added at 100,000 cells on top of each chemotaxis filter. Neutrophil chemotaxis towards PBS (control) or LTB_4_ (10^-6^ M) using a chemotaxis plate after 3 hours was quantified by counting the neutrophils with Neubauer hemocytometer under bright field microscope. Some neutrophils were pre-treated with Rho-Pip-C1 at the indicated concentrations before chemotaxis (n = 5 independent donors in each group, with samples run in duplicate). (B) Quantification of transmigrated neutrophils through fibronectin-coated HUVECs. After 72 hours, human neutrophils were allowed to transmigrate through HUVECs for 3 hours towards PBS (vehicle) or LTB_4_ (10^-6^ M) and were counted using Neubauer hemocytometer. Some neutrophils were pre-treated with Rho-pip-C1 at the indicated concentrations before transmigration (n = 5 independent donors in each group). (C) Neutrophils were treated with vehicle (PBS) or phorbol 12-myristate 13-acetate (PMA) for 3 hours, with and without Rho-pip-C1 (10^-6^ M to 10^-9^ M). MPO levels were then quantified (n = 5 independent donors in each group, with samples run in duplicate) and statistical significance was determined using a one-way ANOVA with Bonferroni post-hoc test (A, C) or Friedman test followed by Dunn's multiple comparison tests between groups (B) and is presented as ^*^p < 0.05 vs. respective PBS control without chemoattractant. All experiments were done in a double-blinded fashion.

An ideal imaging agent to detect neutrophilic inflammation should not only be able to bind with high affinity to the neutrophil, but also not alter the functional response of the cell. To investigate this, we tested the effect of Rho-pip-C1 on human neutrophil chemotaxis using a classic chemotaxis chamber. Figure 3A shows that vehicle-treated neutrophils were able to elicit a chemotactic response to leukotriene B_4_ (LTB_4_, 10^-6^ M) (0.62 ± 0.41 x 10^6^/ml neutrophils vs. 2.79 ± 1.67 x 10^6^/ml neutrophils, PBS vs. LTB_4_ respectively. *p* < 0.05). These responses were not altered by pre-treatment of neutrophils with varying concentrations (10^-6^ M, 10^-7^ M, 10^-8^ M and 10^-9^ M) of Rho-pip-C1, suggesting that the imaging agent does not alter the functional ability of human neutrophils to move towards a chemical gradient.

The ability of neutrophils to extravasate through an endothelial barrier forms a crucial part of the immune response 52. Having determined that Rho-pip-C1 has no effect on neutrophil chemotaxis, we wanted to assess whether the same would hold true when neutrophils moved through an endothelial cell layer towards a chemoattractant. Figure 3B shows that neutrophils pre-treated with 10^-7^ or 10^-8^ M concentrations of Rho-pip-C1 were able to transmigrate through human umbilical vein endothelial cells (HUVEC) towards the LTB_4_ chemoattractant, in a similar fashion to vehicle-treated neutrophils. We found a significant increase (*p* < 0.05) in the number of transmigrated neutrophils within the LTB_4_ groups compared to vehicle (1.60 ± 0.92 x 10^5^/ml neutrophils vs. 5.74 ± 1.17 x 10^5^/ml neutrophils, PBS vs. LTB_4_ respectively. *p* < 0.05. Figure 3B). These results suggest that at certain concentrations, Rho-pip-C1 does not affect the ability of the neutrophil to perform transmigration.

Myeloperoxidase (MPO) is the most abundant pro-inflammatory enzyme stored in the azurophilic granules of neutrophils. Upon stimulation, neutrophils degranulate, releasing their MPO and thereby contributing to the inflammatory environment in a multitude of diseases 53. Rho-pip-C1 had no effect on neutrophil chemotaxis at any concentrations tested and no effect on transmigration at certain concentrations tested (10^-7^ and 10^-8^ M), our final neutrophil functional response assay was to determine whether it had any effect on neutrophil degranulation. Figure 3C shows that all concentrations of Rho-pip-C1 did not alter the ability of neutrophils to produce MPO.

Taken together our data suggest that Rho-pip-C1 does not elicit any additional neutrophil functional responses upon binding to the receptor within the given concentration range and is thus a suitable candidate for *in vivo* tracking of neutrophils.

### Concentration dependent effect of Rho-pip-C1 on neutrophil ROS production

The generation of microbiocidal ROS from activated neutrophils plays an important role in antimicrobial host defence and inflammation. Interestingly, the FPR2/ALX agonist Quin C1 does not induce ROS 34 and might even display inhibitory properties 54, in line with a proposed anti-inflammatory function 34. We assessed ROS levels in Hoxb8-derived murine neutrophils and human neutrophil-like differentiated HL60 cells stimulated for 15 minutes with the lead compound Quin C1 and Rho-pip-C1. Neither compound increased ROS production, whereas both cell types readily responded to control (WKYMVm) stimulation (Figure S7).

### Rho-pip-C1 is a robust fluorescent imaging agent for *in vivo* imaging of acute inflammation

**Figure 4:**
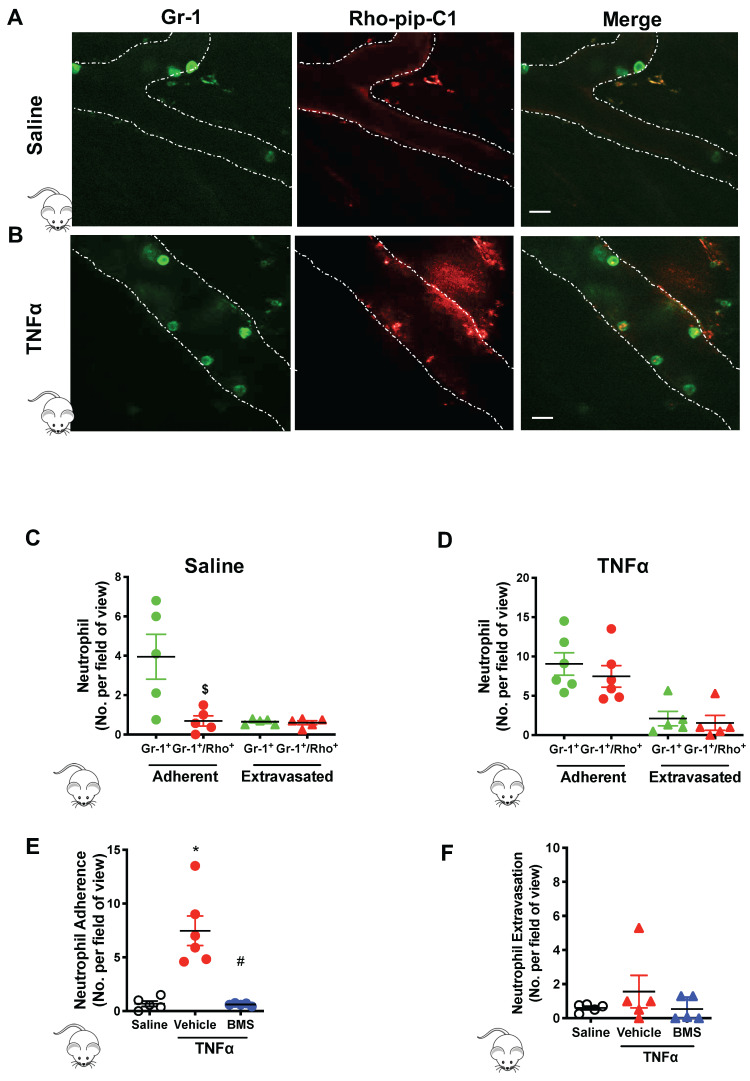
** Preferential uptake of Rho-Pip-C 1 on neutrophils in TNFα treated mice.** Mice were treated with saline (control), TNFα (250 ng in 200 µL saline, intrascrotal injection) or TNFα+BMS-470539 (20 mg/kg via intraperitoneal injection). Confocal intravital microscopy was performed and mice were injected with Gr-1 antibody conjugated with eFluor 488 fluorochrome (green) and Rho-pip-C1 (red). Representative confocal intravital microscopy pictures of cremasteric vessels of (A) saline treated mice and (B)TNFα treated mice. Scale bar, 10 μM. Dotted line represents the edges of the vessel. The number of adherent (within) or extravasated (outside) neutrophils in the cremaster of (C) saline treated (control) (n = 5 mice per group) or (D) TNFα treated (n = 6 mice per group) mice was quantified. Mice were treated with saline (control), TNFα or TNFα+BMS-470539 and the number of (E) adherent (n = 5-6 mice per group) and (F) extravasated neutrophils (n = 5 mice per group) were quantified. Neutrophils were identified by their Gr-1 label and classified as either cells that were positive for Gr-1 (Gr-1^+^. Shown in green) or as cells that had taken up both Gr-1 and Rho-pip-C1 (Gr-1^+^/Rho^+^, dually labelled, shown in red), i.e. neutrophils that had taken up the probe. Statistical significance was determined using unpaired t-test, Mann-Whitney U test (C+D) or ANOVA with Bonferroni post-hoc test (E+F). ^$^*p* < 0.05 vs. Gr-1; ^*^p < 0.05 vs. saline control and ^#^*p* < 0.05 vs. vehicle (saline) + TNFα control. All imaging analysis was done in a double-blinded fashion.

Next, we wanted to determine if Rho-pip-C1 could be used effectively in a pre-clinical disease model of acute inflammation. Mice were injected with saline (vehicle control) or TNFα for two hours. Gr-1 antibody conjugated with eFluor 488 fluorochrome was injected to label the neutrophil population within the cremasteric vasculature, as visualized using confocal intravital microscopy 37. A cohort of mice were also treated with Rho-pip-C1. Neutrophils were identified using the Gr-1 antibody label. In saline treated mice (Figure 4A), there were significantly less neutrophils in the vessels labelled with Rho-pip-C1 (i.e. Gr-1^+^/Rho-pip-C1^+^ cells) than neutrophils labelled with just the Gr-1 antibody (Figure 4C, 3.95 ± 1.14 Gr-1^+^ neutrophils vs. 0.69 ± 0.58 Gr-1^+^/Rho-pip-C1^+^ neutrophils, p < 0.05), whereas no difference between Gr-1^+^/Rho-pip-C1^+^ and Gr-1^+^ neutrophils was observed in extravasated cells (Figure 4C). We also found that a few cells were labelled by Rho-pip-C1 but appeared not to be labelled by the Gr-1 antibody. These cells were Fpr2/ALX+ cells either with unknown origin, or more likely, neutrophils with a very weak Gr-1 signal.

The effectiveness of Rho-pip-C1 as an imaging agent was further confirmed in the TNFα-induced inflammatory response group (Figure 4B), in which over 80% of neutrophils (Gr-1^+^) were also Rho-pip-C1+ (9.05 ± 1.42 Gr-1^+^ neutrophils vs. 7.47 ± 1.37 Gr-1^+^/Rho-pip-C1^+^ neutrophils, Figure 4D). Collectively, these findings suggest that Rho-pip-C1 labels neutrophils in an activated state and is an effective imaging agent to visualize inflammatory events.

To further establish the identity of Rho-pip-C1 as an *in vivo* imaging agent, we treated a cohort of mice with the potent, selective melanocortin 1 (MC_1_) receptor agonist BMS-470539 40. Figure 4E shows that a reduction in the TNFα-induced inflammatory responses elicited by BMS-470539 caused a decrease in the percentage of Rho-pip-C1 tagged neutrophils from 81.68 ± 2.92% to 20.69% ± 9.87% values which were comparable to those observed in the saline control (15.17 ± 5.04 %) 40. The number of labelled, extravasated neutrophils was similar between the saline and TNFα treated mice (Figure 4F).

### Preparation of a pH sensitive analogue for imaging: Compound 13 (Rho-NH-C1)

Having shown that Rho-pip-C1 could be used to image inflammation without changing the functional behavior of neutrophils, we next focused our efforts on developing a “switchable” version (i.e. pH-sensitive) of the compound, which would only 'turn on' in low-pH environments (i.e. as observed in inflammation) 11, 55. To achieve this goal, a rhodamine unit, that is found in its non-emissive spirocyclic form under neutral conditions, was attached to **6** via an ethylenediamine linker (Figure S8). Formation of the adduct Rho-NH-C1 was verified by HRMS and assessed by ^1^H- NMR and ^13^C- NMR (see Supplemental Material for synthetic procedures).

**Figure 5:**
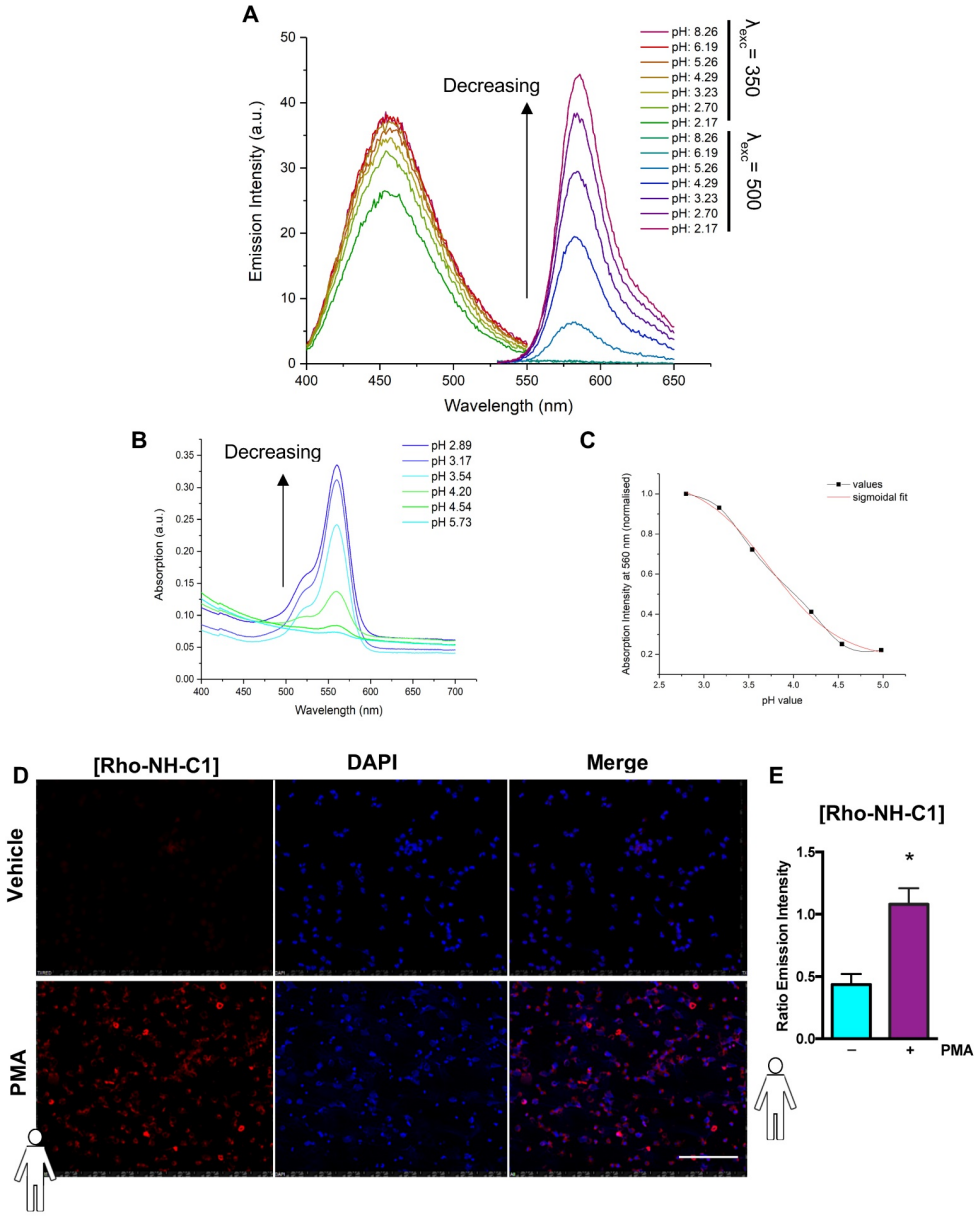
** “Switchable” Rho-NH-C1 is an effective tool for imaging inflammation.** (A) Emission spectra of **13** (Rho-NH-C1. 1 μM, 1:1 v/v methanol and water solutions) at varying pH values (λ_exc_ = 350 nm or λ_exc_ = 500 nm as indicated). (B) Absorption spectra of **13** (33 μM, 1:1 v/v methanol and water solutions) at varying pH values. (C) Determination of pK_cycl_ value using the normalized absorption intensity at 560 nm and a sigmoidal fit (in-built OriginPro 2017 function). (D) Representative images of human neutrophils (n = 5 independent donors) that had been treated with vehicle (saline, tops panels) and phorbol 12-myristate 13-acetate (PMA) (100 nM, bottom panels) prior to incubation with Rho-NH-C1 (scale bar, 100 μM). (E) Quantification of emission intensity ratios demonstrating a statistically significant difference between saline and PMA-treated human neutrophils (n = 5 independent donors in each group). Statistical significance was determined using unpaired t-test and is presented as ^*^p < 0.05 vs. PBS control. All imaging analysis was done in a double-blinded fashion.

A pH-dependent rhodamine emission and absorption profile was observed for Rho-NH-C1 in methanol/water (1:1 v/v) solutions. No rhodamine-centred emission was visible at 581 nm at high pH (values above ~5). Increasing fluorescence and absorbance was observed as the pH value of the solution was decreased (Figure 5A+B). This effect was attributed to the amount of ring-opened form present in solution, with low pH values skewing the spirolactam equilibrium towards the open fluorescent state. The emission band observed at 457 nm for this compound showed no notable pH dependence. In order to determine the intramolecular spirocyclisation equilibrium constant a plot of normalized absorbance at 560 nm against pH value was fitted to a sigmoidal curve function (Figure 5C). Using this method, the pH value at half the maximum absorbance was determined as pK_cycl_ = 3.97. Under normal physiological conditions (pH > 5.5) the tag is expected to remain bound to neutrophils in a predominantly non-fluorescent state, as the rhodamine spirocyclic form prevails. In low pH environments, a fluorescent “on” response is triggered by an equilibrium shift towards the ring-opened rhodamine form resulting in an emission signal.

### *In vitro* assessment of fluorescence response to neutrophil activation

As with Rho-pip-C1, we wanted to test the ability of Rho-NH-C1 to be an effective imaging agent by testing its effect on toxicity and neutrophil functional responses. Rho-NH-C1, as observed with Rho-pip-C1, did not have a toxic effect on HL60 cells or murine neutrophils at any of the concentrations tested (10^-6^ M - 10^-9^ M) (Figure S6). Chemotaxis and transmigration assays were performed in the presence of Rho-NH-C1. At 10^-7^ and 10^-9^ M Rho-NH-C1 elicited no change in neutrophil chemotactic behaviour, whereas all concentrations tested (i.e. 10^-6^ - 10^-9^ M) elicited no statistically significant alteration to neutrophil transmigration, rendering Rho-NH-C1 a useful tool for imaging acute inflammation (Figure S9). In addition, we also found that Rho-NH-C1 did not stimulate FPR2/ALX internalization. Figure 2B shows that Rho-NH-C1 shares a similar profile to that reported for the parent compound, Quin C1, supporting Rho-NH-C1 as a suitable imaging probe. Similarly, as with Rho-pip-C1, at certain concentrations (10^-7^ - 10^-9^ M), Rho-NH-C1 did not activate NADPH oxidase and subsequent superoxide production in human HL60 cells and murine Hoxb8 neutrophils (Figure S7).

### “Switchable” Rho-NH-C1 is an effective tool for imaging inflammation

Finally, to determine whether the “switchable” Rho-NH-C1 could be used in a physiological setting for imaging inflammation, human neutrophils were treated with vehicle (PBS) or PMA and incubated with Rho-NH-C1 or vehicle control. Figure 5D shows that unstimulated Rho-NH-C1 labelled neutrophils were not able to be visualized, although the DAPI stain was clearly visible. In contrast, when the cells were treated with PMA, Rho-NH-C1-labelled neutrophils showed clear, rhodamine-based emission (Figure 5D). Emission intensity was quantified relative to DAPI (Figure 5E) and we demonstrated that the rhodamine-based emission intensity was increased in PMA-treated neutrophils vs. PBS-treated neutrophils (*p* < 0.05), implying that the “switchable” Rho-NH-C1 has the potential to be an effective tool for imaging inflammation.

## Discussion

**Figure 6:**
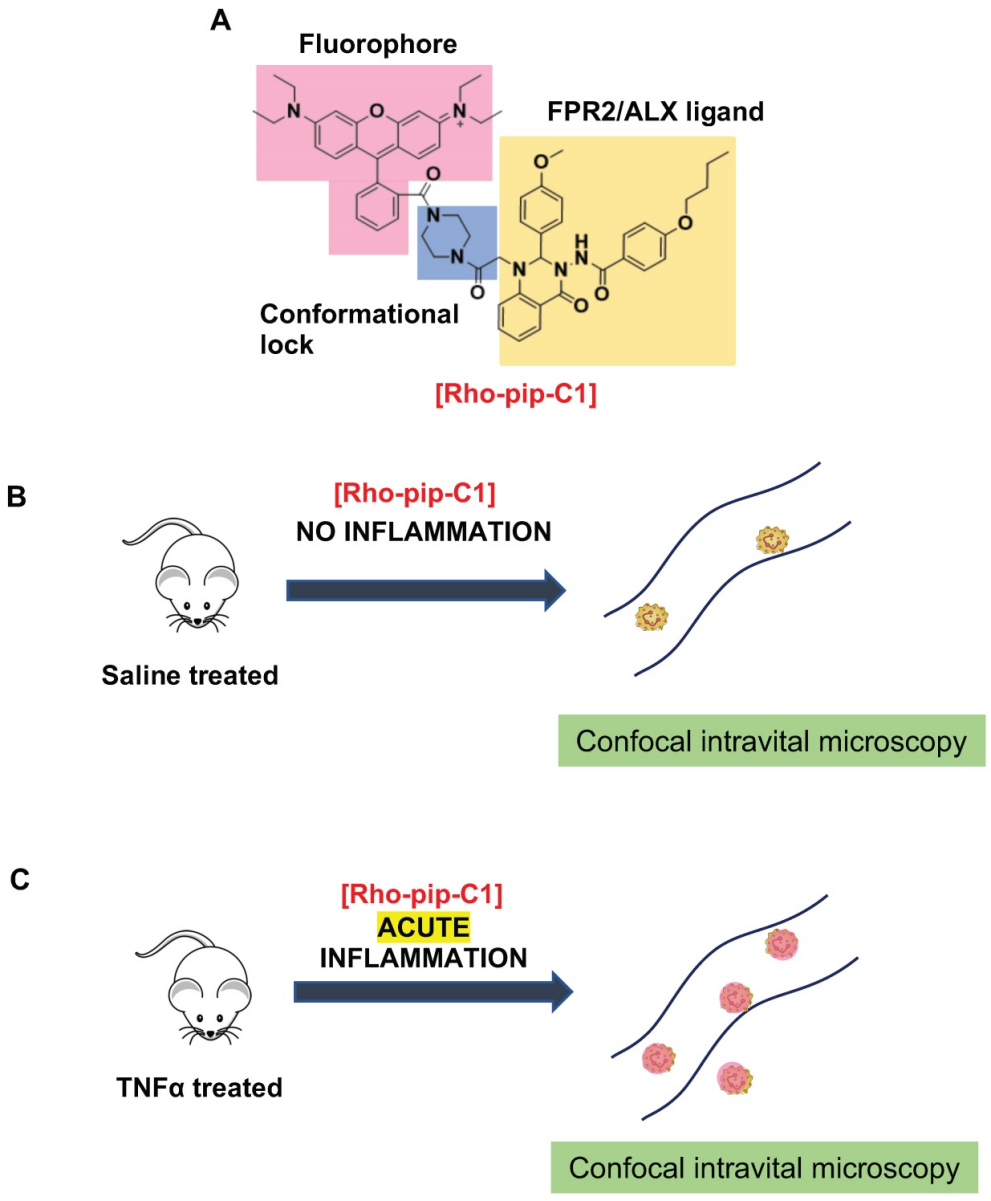
** Design and application of Rho-pip-C1 as a novel small molecule *in vivo* imaging agent for acute inflammation.** (A) Schematic of design elements in FPR2/ALX-targeted fluorescent probe, termed Rho-pip-C1. (B) Under non-inflammatory conditions (i.e. treatment with phosphate buffered saline (vehicle for tumor necrosis factor alpha [TNFα])), Rho-pip-c1 does not bind to FPR2/ALX on neutrophils, therefore no fluorescence is observed in the murine microcirculation using confocal intravital microscopy. (C) Acute inflammation is induced via injection of the pro-inflammatory cytokine TNFα. Rho-pip-C1 binds to FPR2/ALX on activated neutrophils and fluorescence can been observed and quantified using confocal intravital microscopy (recorded at λ_exc_ = 595 nm/ λ_em_ = 645 nm).

Biological processes can be visualized both *in vitro* and *in vivo* using a variety of molecular imaging techniques 12, 56. These techniques allow for their use in monitoring inflammatory disease states (such as tumour environments), for making clinical diagnoses and therapies as well as for biomedical research. Here we have prepared, characterized and tested *in vitro* and *in vivo* a novel FPR2/ALX-targeted small-molecule fluorescent imaging probe, termed Rho-pip-C1 (Figure 6), along with a “switchable” version, termed Rho-NH-C1 for pre-clinical inflammation tracking.

Neutrophils are key players in both driving vascular inflammation and regulating its resolution, making them attractive targets for non-invasive visualization of disease states. The FPR2/ALX receptor plays a central role in both promoting and resolving inflammation and can induce a variety of responses, making this receptor a particular target of interest in biomedical research. To this end, we designed a probe for neutrophil identification and imaging inflammation based on the small-molecule FPR2/ALX ligand motif Quin C1 33, which we have previously demonstrated shows increased uptake in stimulated neutrophils 36. Combination with a brightly fluorescent, rhodamine B- derived fluorophore, which are known for their high extinction coefficients and quantum yields as well as good biocompatibility, allowed us to prepare a fluorescent, FPR2/ALX-targeted probe.

Using pharmacological blocking techniques with the aid of FPR pan antagonist Boc2, we found that Rho-pip-C1 binds to neutrophil FPRs to elicit its signal. This effect was replicated in the presence of the specific FPR2/ALX antagonist WRW4, suggesting an FPR (selectively FPR2/ALX) mechanism for the binding of Rho-pip-C1. In addition, we demonstrated that this binding of Rho-pip-C1 did not induce any changes in receptor internalization or alter neutrophil functional responses (as determined with the use of neutrophil functional assays). These results suggested the suitability of Rho-pip-C1 as an FPR2/ALX imaging probe.

To evaluate these findings in a physiological setting we used an in vivo model of acute inflammation and examined whether our novel probe could be used to visualize key components of the inflammatory cascade i.e. neutrophil adhesion and extravasation. Using confocal intravital microscopy, we found ~80% of identified neutrophils were Rho-pip-C1 positive in the TNFα-treated group, which was substantially higher than previously reported peptide-based FPR probes, where neutrophil labelling *in vivo* has been reported to be in the range of 15% 27. In the saline control group, only 15.17 ± 5.04 % of observed neutrophils were tagged with Rho-pip C1. Thus, Rho-pip-C1 did not just simply act as a neutrophil tag, but also possesses the advantageous quality of preferentially tagging those neutrophils actively involved in the inflammatory cascade (a process characterized by vascular dilation, enhanced permeability of capillaries, increased blood flow and leukocyte recruitment, all of which could increase the uptake of the dye)^57^. These effects were further confirmed by the visualization and quantification of extravasated Rho-pip-C1^+^ neutrophils in both the saline and the TNFα group. Upon attenuation of the inflammatory response using BMS 470539, the number of labelled cells within the vessel was comparable to what was observed in the saline control group, whereas extravasated neutrophils were Rho-pip-C1 labelled. These results suggest that Rho-pip-C1 could be used as an imaging agent to successfully quantify the effect of an anti-inflammatory drug for drug discovery programmes.

Inflammation is known to be associated with a lowered local extracellular pH range and the resulting acidification is central in the regulation of neutrophil functions 11, 55. Accordingly, sensors that are bound to neutrophils in a non-fluorescent state and only selectively flag up neutrophils as they enter into an acidic environment through a fluorescent “on” response have the advantage that their switchable signal could relay valuable, real-time readouts on inflammatory status and cellular activity. Therefore, a pH-responsive rhodamine form was combined with Quin C1 to afford Rho-NH-C1. Our novel probe was deemed to be almost non-fluorescent at neutrophil pH and highly fluorescent at acidic pH, as determined by the emission intensity increasing as the pH was decreased. These effects were further confirmed by the ability of Rho-NH-C1 to produce a fluorescent signal with PMA-activated neutrophils, but not neutrophils treated with the vehicle, in which the rhodamine unit remained in a non-fluorescent state. Future studies will determine whether Rho-NH-C1 and/or Rho-pip-C1 may act as an ago-allosteric modulator of FPR2/ALX in a similar fashion to the parent compound Quin C1.

## Conclusion

We have developed a novel small molecule FPR2/ALX imaging probe which lacks the complications associated with previous FPR imaging probes (i.e. target cell internalization and activation) and binds to activated neutrophils (i.e. those involved in the activation of the inflammatory cascade), thereby differentiating it from previous FPR-probes. Additionally, simple synthetic modifications enabled us to capitalize on our imaging probe and make a “switchable” analogue (Rho-NH-C1), providing a promising basis as a sensor of inflammation for biomedical research and a screening tool for drug discovery.

## Supplementary Material

Supplementary figures and tables.

## Abbreviations

ALX: lipoxin receptor
ANOVA: analysis of variance
BOC: *tert*-Butyloxycarbonyl
Boc2: *N*-*tert*-butyloxycarbonyl-Phe-Leu-Phe-Leu-Phe
cFLFLF: cinnamoyl-Phe-D-Leu-Phe-D-Leu-Phe-Lys-NH_2_
DAPI: 4',6-diamidino-2-phenylindole
DHR: dihydrorhodamine
DMEM: Dulbecco's modified eagle medium
EDTA: 2,2',2'',2'''-(Ethane-1,2-diyldinitrilo)tetraacetic acid
fMLF: *N*-formyl-methionyl leucyl phenylalanine
FPR: formyl peptide receptor
GPCR: G protein-coupled receptors
Gr-1: granulocyte receptor-1 antigen
HL60: human leukemia cell line 60
Hoxb8: homeobox protein B8
HRMS: high resolution mass spectrometry
HUVEC: human umbilical vein endothelial cells
IgG: immunoglobulin G
ip: intraperitoneal
IVM: intravital microscopy
LTB_4_: leukotriene B4
MALDI: matrix assisted laser desorption ionisation
MRI: magnetic resonance imaging
MPO: myeloperoxidase
NIR: near-infrared
NMR: nuclear magnetic resonance
PBS: phosphate-buffered saline
PEG: polyethylene glycol
PET: positron emission tomography
PMA: phorbol 12-myristate 13-acetate
qPCR: quantitative polymerase chain reaction
Quin C1: -butoxy-N-[2-(4-methoxy-phenyl)-4-oxo-1,4-dihydro-2H-quinazolin-3-yl]-benzamide
ROS: reactive oxygen species
RPMI: Roswell park memorial institute medium
SEM: standard error of mean
SPECT: single-photon emission computed tomography
TMB: 3,3',5,5'-tetramethylbenzidine
TNFα: Tumor necrosis factor-alpha
WKYMVm: Trp-Lys-Tyr-Met-Val-D-Met-NH_2_
WRW4: Trp-Arg-Trp-Trp-Trp-Trp
