# Supplementary material for: Targeting of Formyl Peptide Receptor 2 for in vivo imaging of acute vascular inflammation
Source: Theranostics. 2020 May 17;10(15):6599–614. doi: 10.7150/thno.44226 (PMC7295040; doi:10.7150/thno.44226)
Supplement: Supplementary file 1 — Supplementary figures and tables. [file thnov10p6599s1.pdf]

Supplementary Material

Targeting of Formyl Peptide Receptor 2 for *in vivo* imaging of acute vascular inflammation

Authors: Tamara Boltersdorf[1], Junaid Ansari[2,3], Elena Y. Senchenkova[2], Jieny Groeper[4], Denise Pajonczyk[4], Shantel A. Vital[2], Gaganpreet Kaur[2], J. Steve Alexander[2], Thomas Vogl[5], Ursula Rescher[4], Nicholas J. Long[1#], Felicity N. E. Gavins[2,6#*]

Affiliations:

[1]Department of Chemistry, Imperial College London, Molecular Sciences Research Hub, White City, London, W12 0BZ, UK.

[2]Department of Neuroscience, Louisiana State University Health Sciences Center-Shreveport, Shreveport, LA, 71130, USA.

[3]Department of Molecular & Cellular Physiology, Louisiana State University Health Sciences Center-Shreveport, Shreveport, LA, 71130, USA.

[4]Institute of Medical Biochemistry, Centre for Molecular Biology of Inflammation, University of Muenster, D-48149 Muenster, Germany.

[5]Institute for Immunology, University of Muenster, D-48149 Muenster, Germany.

[6]Department of Life Sciences, Brunel University London, Uxbridge, Middlesex, UB8 3PH, UK.

[#]Authors contributed equally

Short Title: Rho-pip-c1 flags FPR2/ALX dependent vascular inflammation

*Corresponding author:

Felicity N. E. Gavins

Department of Life Sciences

Brunel University London

Kingston Lane

London

Uxbridge

UB8 3PH

Tel: +44 (0) 1895 267 151

Email: felicity.gavins@brunel.ac.uk

Supplementary Material and Methods

Synthetic Procedures

Methyl 4-butoxybenzoate (**2**), 4-butoxybenzohydrazide (**3**), 2-(4-butoxybenzoyl)hydrazide 2-nitro-benzoic acid (**4**), 2-(4-butoxybenzoyl)hydrazide 2-amino-benzoic acid (**5**) and 4-butoxy-*N*-[1,4-dihydro-2-(4-methoxyphenyl)-4-oxo-3(2H)-quinazolinyl]-benzamide (Quin C1, **6**) were prepared and purified according to protocols modified from reference.[1]

Compound 10

Rhodamine B piperazine amide (1.00 g, 2.0 mmol) was prepared according to literature procedures[2] and dissolved in dichloromethane (30 mL) at 0°C. Triethylamine (1.11 mL, 8.0 mmol) was added to the mixture followed by dropwise addition of chloroacetyl chloride (0.18 mL, 2.2 mmol). The solution was stirred and slowly warmed to room temperature. After 3 hours water was added and the aqueous phase was extracted with dichloromethane. Organic fractions were combined and dried over magnesium sulphate prior to separation of the desired product by silica column chromatography eluting in dichloromethane with an acetone gradient, to yield a pink solid. (0.46 g, 39%).

^{1}H-NMR (400 MHz, d6-acetone), δ_H (ppm): 1.33 (12H, t, $^{3}J_{HH}$ = 7.9 Hz), 3.24-3.67 (8H, br), 3.77 (8H, qu, $^{3}J_{HH}$ = 7.1 Hz), 4.23 (2H, br s), 6.95 (2H, br m), 7.17 (2H, br), 7.35 (2H, d, $^{3}J_{HH}$ = 9.5 Hz), 7.54-7.60 (1H, m), 7.73-7.82 (3H, m). ^{13}C-NMR (400 MHz, d6- acetone) δ_H (ppm): 12.8, 38.7, 42.0, 46.6, 96.9, 114.6, 115.1, 128.6, 130.7, 131.3, 132.1, 133.1, 156.7, 158.7. MS *m/z*: 587 {M}$^{+}$.

Compound 11

To a solution of rhodamine B piperazine amide acetyl chloride (0.012 g, 20 µmol) in acetonitrile (5 mL) N,N-diisopropylethylamine (18 µL) and Quin C1 (0.010 g, 22 µmol) were added and the mixture was stirred overnight at 65°C. Purification was achieved by repeated silica column chromatography, initially eluting in dichloromethane and acetone (10%), with an added gradient of methanol (up to 30%). Subsequently, the fractions containing the desired product were separated again using a dichloromethane/acetone (4:1) eluent. The product was obtained as a deep pink solid (0.006 g, 30%).

^{1}H-NMR (400 MHz, acetone-d6), δ_H (ppm): 0.99 (3H, m) 1.06-1.40 (12H, m), 1.51 (2H, m), 1.77 (2H, m), 3.12- 4.18 (23H, br), 6.10-8.61 (22H, br). ^{13}C-NMR (500 MHz, d6- acetone) δ_H

(ppm), spectrum faint: 11.7, 12.8, 14.4, 19.8, 23.3, 23.7, 32.6, 33.9, 35.2, 37.6, 41.2, 46.6, 64.5, 66.2, 66.5, 73.4, 97.0, 113.4-116.1 several peaks, 129.3, 130.6, 132.0, 133.5, 135.6, 156.8, 157.1, 158.7, 168.0. HRMS *m/z*: found: 996.5035 calculated: 996.5024.

Compound 13

Rhodamine ethylenediamine, prepared according to literature procedures[3] (0.011 g, 22 μmol), 4-butoxy-N-(1-(2-chloroacetyl)-2-(4-methoxyphenyl)-4-oxo-1,4-dihydroquinazolin-3(2H)-yl)benzamide (0.010 g, 18 μmol) and potassium carbonate (0.015 g, 109 μmol) were stirred in acetonitrile (2 mL) at 60°C for 17 hours. The product was isolated by silica column chromatography eluting in a dichloromethane and acetone mixture (9:1) as a pink solid (0.011g, 63%).

^{1}H-NMR (400 MHz, acetone-d6), δ_H (ppm): 0.97 (3H, t, $^3J_{HH}$= 7.4 Hz) 1.12 (12H, t, $^3J_{HH}$= 7.1 Hz), 1.50 (2H, m), 1.77 (2H, m), 2.50- 2.64 (2H, m), 3.26- 3.42 (10H, br), 3.72 (3H, s), 3.84-4.01 (2H, br), 4.08 (2H, t, $^3J_{HH}$= 6.5 Hz), 6.32-6.54 (6H, m), 6.80 (2H, d), 6.98-7.06 (3H, m), 7.30 (1H, m, br), 7.41 (2H, m, br), 7.47- 7.56 (4H, m), 7.77-8.05 (4H, m), 10.32 (1H, br). HRMS *m/z*: found: 970.4863 calculated: 970.4867.

4-Butoxy-N-(1-(2-chloroacetyl)-2-(4-methoxyphenyl)-4-oxo-1,4-dihydroquinazolin-3(2H)-yl)benzamide (14)

Quin C1 (0.10 g, 0.22 mmol) and triethylamine (0.03 g, 0.33 mmol) were dissolved in dichloromethane (3 mL) under a nitrogen atmosphere. The stirring solution was immersed in an ice/acetone bath and chloroacetyl chloride (0.03 g, 0.26 mmol) in dichloromethane (2 mL) was added dropwise. The reaction was gradually warmed to room temperature and stirred overnight. The crude mixture was washed with water (5 mL) and dried over magnesium sulphate. Purification was achieved by silica column chromatography eluting with a dichloromethane/acetone mixture (95:5) to afford an off-white solid (0.07 g, 60%).

^{1}H-NMR (400 MHz, d6- acetone) δ_H (ppm): 0.97 (3H, t, $^3J_{HH}$= 7.4 Hz), 1.50 (2H, m), 1.78 (2H, m), 3.72 (3H, s), 4.08 (2H, t, $^3J_{HH}$= 6.5 Hz), 4.84 (2H, br), 6.81 (2H, dt, $^3J_{ortho}$= 8.9 Hz, $^4J_{meta}$= 2.1 Hz,), 7.02 (2H, m), 7.13 (1H, br, s), 7.39 (3H, m), 7.58 (2H, m), 7.96 (3H, m), 10.37 (1H, br). Exact assignment aided by NOESY spectroscopy. ^{13}C-NMR (400 MHz, d6- acetone) δ_H (ppm): 13.1, 18.8, 30.9, 54.5, 67.6, 113.7, 114.1, 124.0, 126.4, 127.7, 128.5, 129.6, 132.9, 159.8, 160.4, 162.5, 165.7. HRMS: *m/z* calculated: 552.1790, found: 552.1793 for $\{M+H\}^+$.

Supplemental Figures and Tables

Figure S1: LC chromatogram of compound **6**.

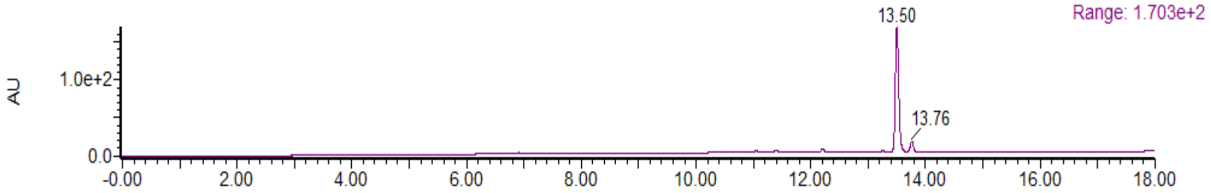

Figure S2: LC chromatogram of compound **11**

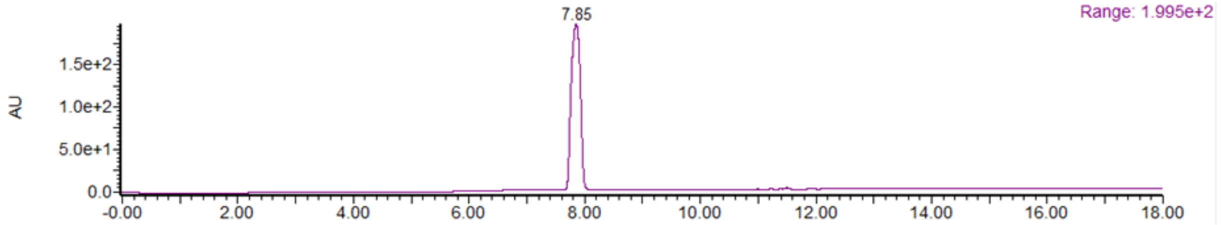

.

Figure S3: LC chromatogram of compound **13**.

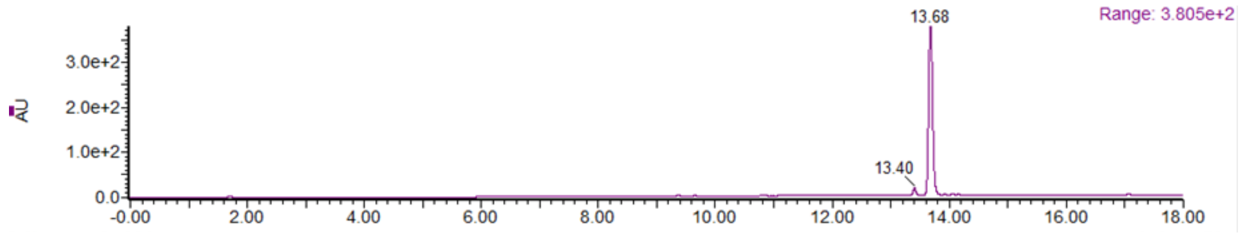

Figure S4: LC chromatogram of compound **14**.

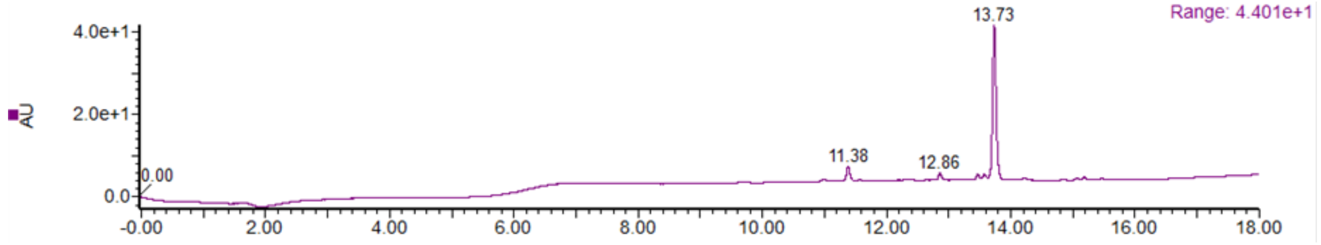

Figure S5: Excitation (red, λ_{em}=580 nm) and emission spectra (blue, λ_{exc}= 350 nm) of 11 in methanol.

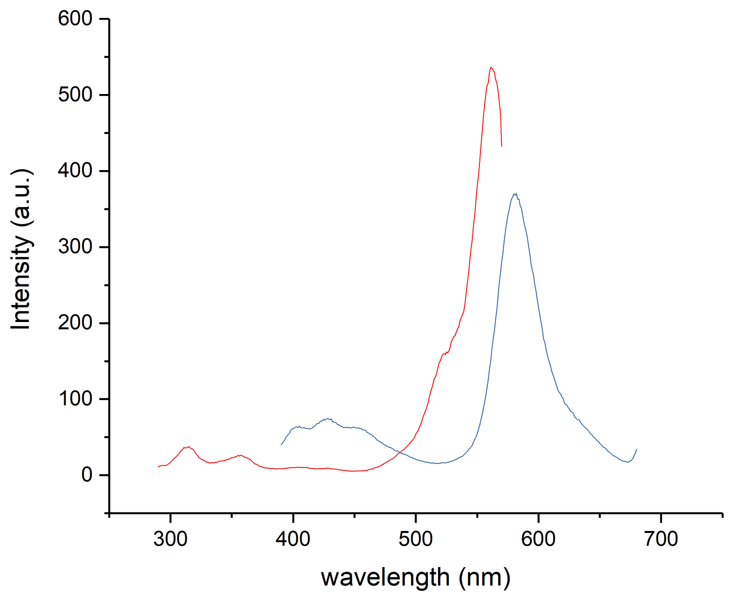

Figure S6: Analysis of agonist-induced cytotoxicity.

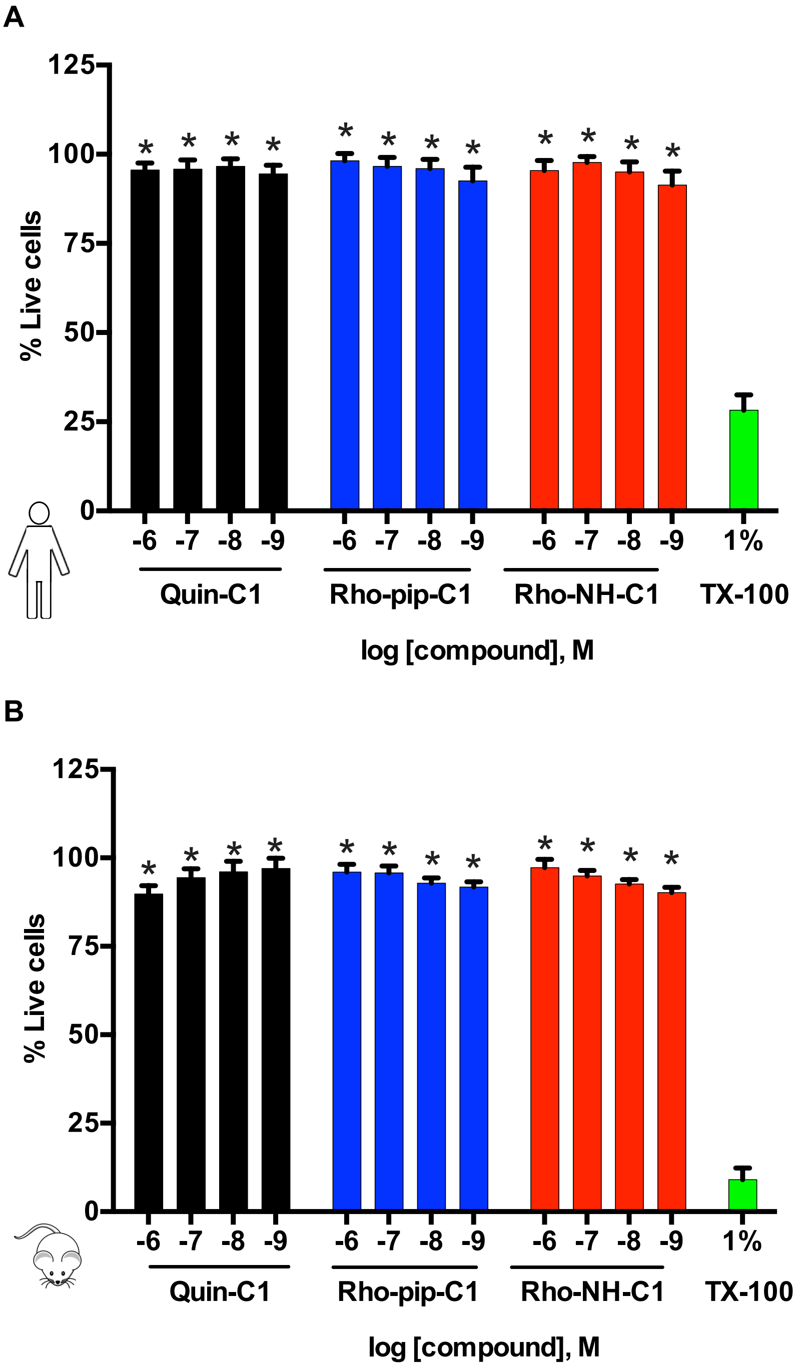

(A) Human HL-60 cells differentiated toward neutrophils or (B) murine neutrophils generated from Hoxb8 neutrophil progenitor cells were stimulated with the indicated agonists. Numbers of live cells and compromised cells were quantified after 15 minutes by flow cytometry and are expressed as % of total cells (10.000 cells/condition). n = 6 independent experiments in each group. $^{*}p < 0.05$ vs. TX-100 group.

Figure S7: Analysis of agonist-induced reactive oxygen species (ROS) generation.

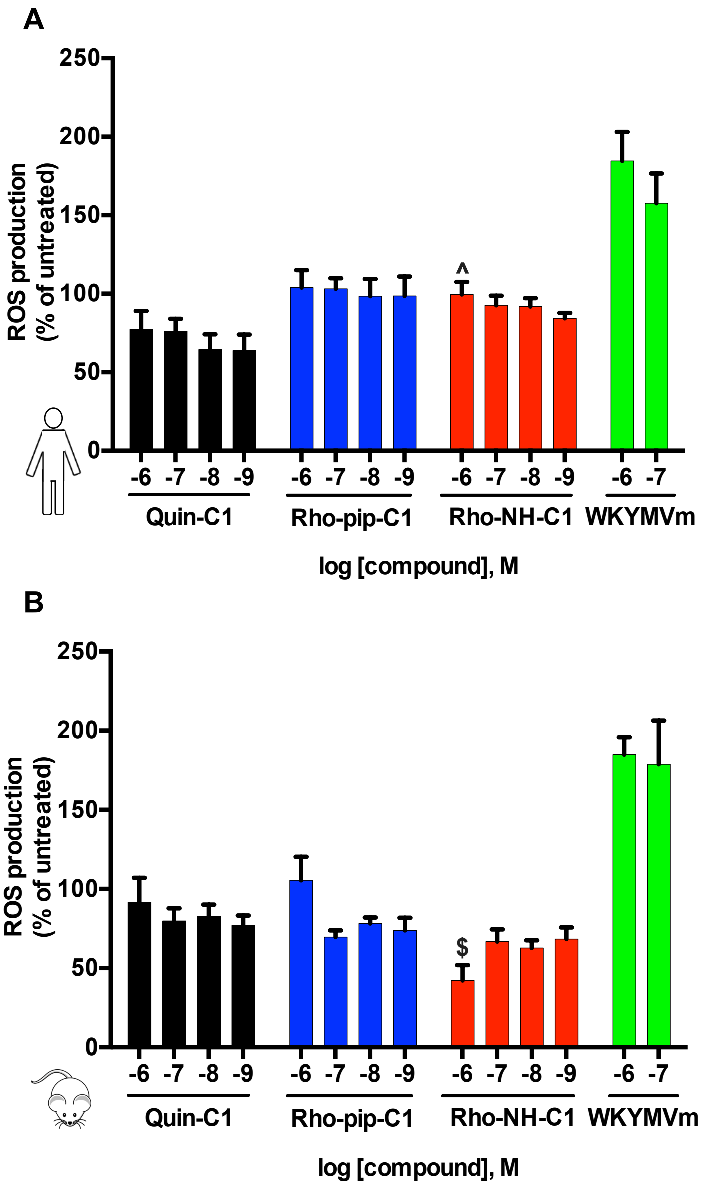

(A) Human HL-60 cells differentiated toward neutrophils or (B) murine neutrophils generated from Hoxb8 neutrophil progenitor cells were stimulated with the indicated agonists. ROS production was analysed after 15 minutes by flow cytometry and is compared to the signal detected in untreated cells (100%). n = 5 independent experiments in each group for HL-60 cells and n = 6 independent experiments for murine Hoxb8 neutrophils. $^{\$}p < 0.05$ vs. Quin-C1 [10^{-6} M], $^{\wedge}p < 0.05$ vs. Quin-C1 [10^{-9} M].

Figure S8: Synthetic pathway for preparation of compound 13.

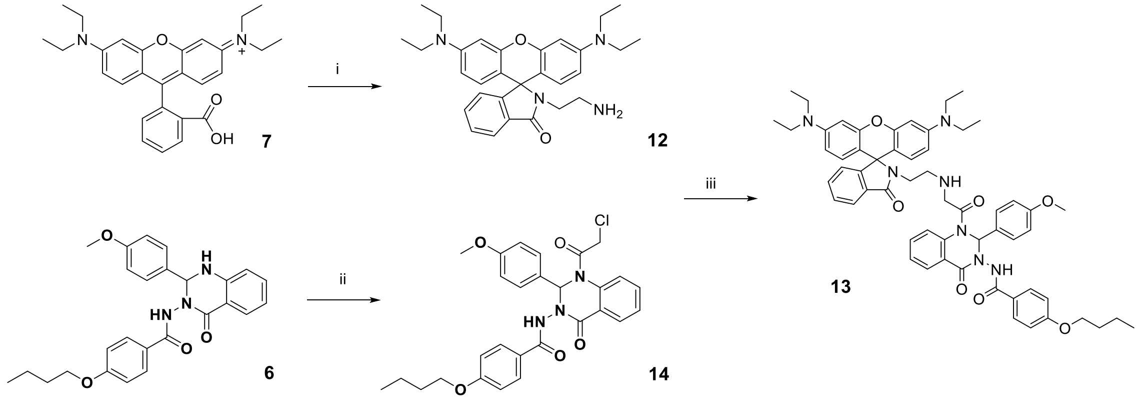

Reagents: i) ethylenediamine (excess), ethanol, ii) chloroacetyl chloride, triethylamine, dichloromethane, iii) **14**, potassium carbonate, acetonitrile.

Figure S9: Rho-NH-C1 does not elicit changes in neutrophil function.

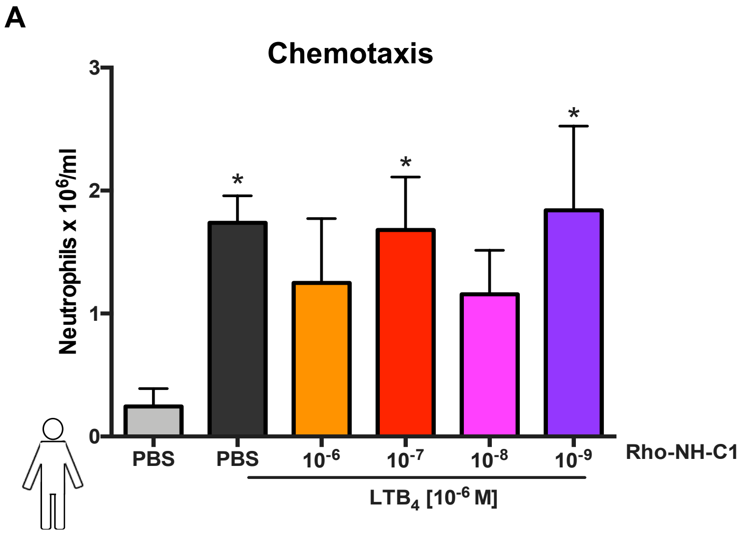

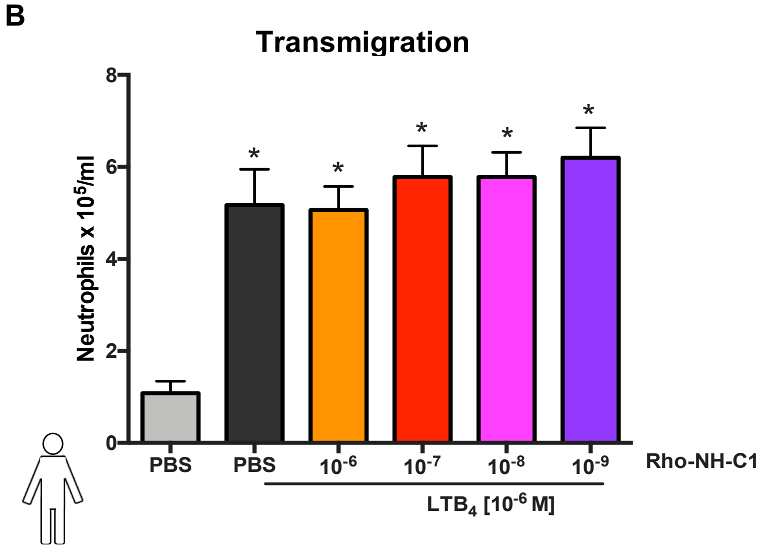

(A) Neutrophils isolated from peripheral blood of healthy volunteers were resuspended in DMEM with 3% FBS and added at 100,000 cells on top of each chemotaxis filter. Neutrophil chemotaxis towards PBS (control) or LTB_4 (10^{-6} M) using a chemotaxis plate with a 3 µM pore size after 3 hours was quantified by counting the neutrophils with Neubauer hemocytometer under a brightfield microscope. Some neutrophils were pre-treated with Rho-NH-C1 at the indicated concentrations before chemotaxis (n = 5 independent donors in each group, with samples run in triplicate). (B) Quantification of transmigrated neutrophils through HUVECs. HUVECs were cultured on fibronectin-coated 8 µM pore size polyester membrane sterile

inserts. After 72 hours, human neutrophils were allowed to transmigrate through HUVECs for 3 hours towards PBS (control) or LTB_4 (10^{-6} M) and were counted using a Neubauer hemocytometer. Some neutrophils were pre-treated with Rho-NH-C1 at the indicated concentrations before transmigration (n = 5 independent donors in each group). Statistical significance was determined using a one-way ANOVA with Bonferroni post-hoc test (A) or Friedman with Dunn's test (B) and presented as $^{*}p < 0.05$ vs. PBS control.

Table S1: Past and current (IUPHAR) nomenclature of the Formyl Peptide Receptors (FPRs).

Species	Current (IUPHAR) Nomenclature	Previous Nomenclature
Human	FPR1	Formyl-peptide receptor 1
		FPR
		FMLPR
		NFPR
	FPR2	Formyl-peptide receptor like 1
		FPRL1
		Lipoxin A_4 receptor
		ALXR
		FPR2A
		FMLPX
		FPRH1
		RFP
		HM63
	FPR3	Formyl-peptide receptor like 2
		FPRL2

		**fMLP-related receptor 1**
		**FPRH2**
		**FMLPY**
**Mouse**	**Fpr1**	**Fpr1**
	**Fpr2**	**Fpr-rs2**
	**Fpr3**	**Fpr-rs3**
		**fprL1**
		**mALXR**

References:

1. Boltersdorf T, Ansari J, Senchenkova EY, Jiang L, White AJP, Coogan M, et al. Development, characterisation and in vitro evaluation of lanthanide-based FPR2/ALX-targeted imaging probes. Dalton Trans. 2019; 48: 16764-75.
2. L. Bi: Novel probes and targeting compounds for mitochondria, patent 2014, WO2014063033 (A2).
3. Zhang X, Shiraishi Y, Hirai T, Cu(II)-Selective Green Fluorescence of a Rhodamine−Diacetic Acid Conjugate, Org. Lett. 2007; 9: 5039–42.
